# Might as Well Jump: Sound Affects Muscle Activation in Skateboarding

**DOI:** 10.1371/journal.pone.0090156

**Published:** 2014-03-11

**Authors:** Paola Cesari, Ivan Camponogara, Stefano Papetti, Davide Rocchesso, Federico Fontana

**Affiliations:** 1 Department of Neurological and Movement Sciences, University of Verona, Verona, Italy; 2 Department of Mathematic and Computer Science, University of Udine, Udine, Italy; 3 Department of Architecture and Arts, Iuav University of Venice, Venice, Italy; 4 Institute for Computer Music and Sound Technology, Zurich University of the Arts, Zurich, Switzerland; University of Rome, Italy

## Abstract

The aim of the study is to reveal the role of sound in action anticipation and performance, and to test whether the level of precision in action planning and execution is related to the level of sensorimotor skills and experience that listeners possess about a specific action. Individuals ranging from 18 to 75 years of age - some of them without any skills in skateboarding and others experts in this sport - were compared in their ability to anticipate and simulate a skateboarding jump by listening to the sound it produces. Only skaters were able to modulate the forces underfoot and to apply muscle synergies that closely resembled the ones that a skater would use if actually jumping on a skateboard. More importantly we showed that only skaters were able to plan the action by activating anticipatory postural adjustments about 200 ms after the jump event. We conclude that expert patterns are guided by auditory events that trigger proper anticipations of the corresponding patterns of movements.

## Introduction

Humans are able to recognize and discern among different types of events just by listening to the sounds they produce. It is easier to recognize the action that gives rise to the event (i.e., scraping, breaking) rather than the object properties on which the same event is targeted such as its surface or the material it is made of [1]–[5]. Many everyday sounds trigger intentions for moving, as it happens for instance when a telephone is ringing. It would be relevant to understand if and how these sounds influence the performance of actions. Recently it has been shown that during fast hand grasping, the kinematics of the grip changed depending on the congruence of a sound simulating the same action [6], [7], suggesting the presence of associations between sound perception and action planning. An interesting implication is that, during the perception of sound events, both behavioral and neuronal properties might be involved in a common mechanism. Neural imaging studies have shown that the sounds of actions in particular those produced by human gestures activate motor and pre-motor areas, whereas sounds of different nature, such as noise and environmental sound, do not [8]. The neuronal processes underlying action sound recognition produced convincing evidence that the same populations of neurons are active both when listening to an action and when performing it [4], [9]. This evidence calls for the presence of a mirror neuron system not just for action execution and (visual) observation, but also for its auditory perception [2], [10]. However, the degree of motor activation registered in presence of auditory events strongly depends on the level of familiarity and motor experience that the listener has with the related action [11]–[13]. After a period of training for performing a sequence of notes on a piano, non-musicians showed far greater activity in their motor system when they were listening to the learned sequence as compared to that obtained by reversing the order of the notes [14]. Given the existence of a neuronal network refined by auditory-motor occurrence, it could be possible that an auditory event is sufficient to recruit motor activations that provide the basis for movement execution and anticipation [15]–[17]. Recently it has been shown that this is the case. Individuals demonstrated the ability to perceive spatial and temporal attributes of walking by relying on audition alone [18], [19]. If movement patterns are embedded in an auditory percept and hence integrated into a motor scheme, then this scheme must anticipate also the corresponding patterns of movement.

Theoretical models indicate that the human motor system is designed to act as an anticipatory mechanism and that humans are able to pre-plan forthcoming actions through an internal forward model [20]–[23]. Indeed several TMS and fMRI studies support the existence of an internal simulation of an action evoked by the sound it produces [24]–[26]. This internal model has been shown to be involved in planning and predicting even complex actions [27], [28] by underlying the relevance of cross-modal integration between action perception and execution [29]–[31].

From a neurophysiological point of view, anticipatory behaviors are defined by the presence of Anticipatory Postural Adjustments (APAs) in the entire body [32]. APAs have been introduced to address the shifts of the body center of pressure seen prior to the initiation of a voluntary motor action [33]. They represent changes in the background activity of muscles and the associated shift of the Center of Pressure (COP) detected prior to the initiation of a voluntary movement [34]. They appear before an active fast movement in the postural muscles and are measured as onset and amount of muscle activation [35]–[38]. The purpose of APAs is to counterbalance the mechanical effect of expected perturbations for maintaining individual equilibrium [38], and their modulation strongly depends on the level of motor capabilities expressed by an individual [39]–[41]. From this perspective APAs represent *ad hoc* measures for obtaining a deeper understanding about the role that auditory information play in action preparation and execution, by testing people representing different movement skill levels. Evidences about the existence of anticipatory mechanisms triggered by auditory stimuli are very limited and no study, to our knowledge, has yet considered APAs modulation under the presence of expected sound perturbation.

The focus of this study is to reveal the role of sound in action anticipation and performance, and to test whether accuracy in planning and executing is related to the level of sensorimotor experience that listeners have about a specific action. The main aim is to search for the presence of an internal movement simulation evoked by the sound it produces, and revealed by specific neuromuscular activations.

Here, a sequence of postures elicited by hearing the sound of rolling wheels is studied. Participants were exposed to synthetic vibro-acoustic feedback underfoot, as if they were riding a skateboard along a prescribed path. We tested the performance of individuals ranging from 18 to 75 years of age, some of them without any skill in skateboarding and others instead being experts in this sport. Biomechanical and muscular measurements were collected to assess the presence of APAs: through the use of force platforms we measured the subjective forces acting underfoot, and through EMG measurements we extracted the onset times and the amount of muscular activation while experiencing virtual skateboarding. We also collected and analyzed the subjects' impression about the perceived experience, expressed as hand-drawn altimetric traces.

## Materials and Methods

### Ethics Statement

The experimental protocol received a written approval by the members of the Ethics Committee of the Department of Neurological and Movement Sciences of the University of Verona. All participants provided their written informed consent prior to entering the study, which had been also approved by the institutional review board.

### Participants

Twenty participants were recruited and divided into three groups: six old adults (mean age 69.5±4.62 years, mean weight 79.16±8.95 Kg) six young adults (mean age 20.25±1.9 years, mean weight 65.80±12.47 Kg) and eight expert skaters (mean age 19.6±2.73 years, mean weight 66.37±6.20 Kg). Expert skaters had to have at least 3 years of practice and training for at least twice a week. All participants presented neither muscle skeletal, nor neurological impairments. Foot dominance was assessed [42]. The main aim in recruiting non-expert old and young adults groups was to test whether the performance was related to motor skills and age.

### Apparatus

**Figure 1:**
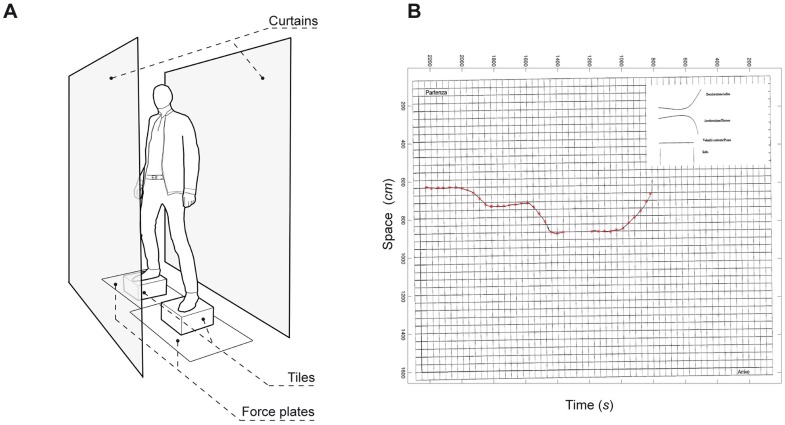
Experimental setup. A) Setup, B) Typical altimetry profile obtained from one individual.

Six electrodes (EMG-Zero-wire system) were applied on six muscles, three for each side of the body: the Gastrocnemius (G), the Tibialis Anterior (TA) and the Rectus Femoris (RF) [43]. Two force platforms (AMTI & Kistler) were employed to record the Center of Pressure (COP) migration and the force vector components Fx, Fy, Fz at a sampling frequency of 1200 Hz. Two vibro-acoustic transducers (Clark Synthesis TST239) were screwed each below a 30×30 cm wooden tile. Either tile was finally placed on the respective force platform. A curtain surrounded the area for enclosing the experimental setup (Figure 1A).

### Stimuli

Three sound stimuli having the same time length (30.8 s) were prepared: brown noise, which was used for control purposes along with silence, and two synthetic sound sequences (Sound S1 and Sound S2) simulating the run of a skateboard along a virtual path. The auditory path exposed typical events that occur during skateboarding: acceleration, steady run, deceleration, and jump. By realizing a different composition of such events, either sound sequence then defined its own path. The auditory paths were synthesized using a physically based model providing rolling sounds depending on several interactive control parameters, including the speed of rolling [44]. The stimuli were finally reproduced by the vibro-acoustic transducers located underfoot.

### Procedure

Participants stood on the tiles, orienting their dominant foot forward and the other foot backward as in a typical skateboarding posture. In the first control task silence was made, and they repeated three trials lasting 40 seconds in which they were asked to stand still. In the second control task they performed three trials, by standing still while hearing brown noise through headphones. Finally they were asked to perform a skateboarding action, in the limits of their skills, along 30 listening trials to the two auditory paths. There were two blocks made of 15 trials each. After the 2^nd^ and the 15^th^ trial participants were asked to step down from the tiles and profile the path they had experienced on an altimetry grid, according to the prescribed directions (Figure 1B, see Appendix S1 for detail). Finally, both control tasks were repeated before the end of the test (Table 1 summarize the whole experimental procedure).

**Table 1:** Experimental procedure.

Experimental conditions	# trials
Control (no sound)	3
Control (noise)	3
Skateboard sound	2
Drawing test	1
Skateboard sound	13
Drawing test	1
Skateboard sound	2
Drawing test	1
Skateboard sound	13
Drawing test	1
Control (no sound)	3
Control (noise)	3

All conditions are in a chronological order.

### Processing of the responses

**Figure 2:**
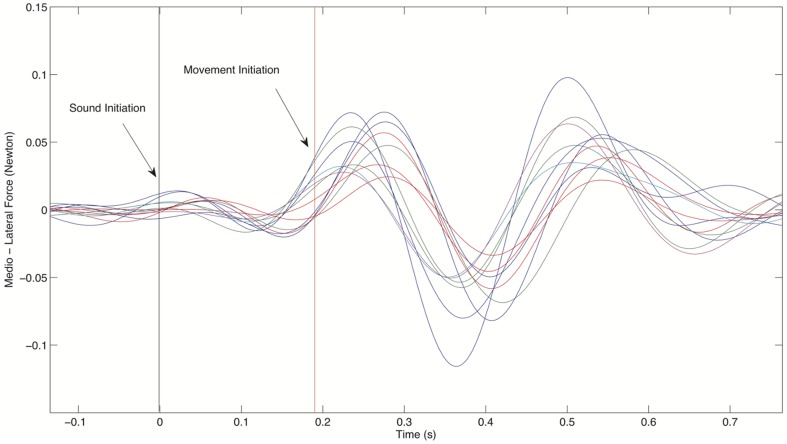
Several trials obtained by one skater participant showing the anterior-posterior force profile in Newton (in the y axis) over time (in the x axis). Time zero t_0_ represents the sound of the jump initiation; the red vertical line represents the mean detection for the force changes over trials.

From either auditory path, a sufficiently large temporal window was set around the jump event. For each trial, synchronized EMG and force signals were selected exactly in this window by aligning them altogether with respect to the jump event. This event, in fact, could be accurately isolated in all recorded signals since being free of exogenous energy conveyed by the vibro-acoustic transducers in the audio band, ranging approximately from 35 to 16000 Hz. Conversely, outside the jump event the transducers were active and consequently energy in the audio band was delivered to the force platforms; however, its spectral content did not overlap with the subjects' exerted force signals, whose spectral energy was instead located below 20 Hz. Hence, the force could be easily isolated through low-pass, zero-lagged digital filtering of the force signals. To quantify the presence of APAs, the early changes in Fx and Fy, i.e. the forces respectively along the antero-posterior and latero-lateral direction, were defined as the difference between the baseline value and the maximum peak value of the signal in the range [t_0_−300, t_0_+300] ms, in which t_0_ is the time when the jump event starts [3]. These values were then normalized by the respective subject's weight. Additionally, the onset of the same forces was defined as the time when Fx and Fy deviated from their respective baseline by 5% of their peak (Figure 2). To quantify the movement strategy adopted during the jump, the time to peak and amplitude of all normal forces (Fz) were considered on both platforms.

Similarly to the force, the EMG signals were time-aligned in correspondence of the jump event. The EMG signals were first full-wave rectified and filtered with a 100 Hz low-pass, fifth order Butterworth filter. Later to detect the onset of the EMG signal, we run a 10 Hz low-pass, fifth order Butterworth filter. To detect the onset of EMG activity (t_0_), we considered the first deflection above two standard deviations from its baseline. The level of baseline was defined as the mean EMG activity within a window time delimited by the sound of the jump initiation and considering 400 ms before that instant [38], [39]. To quantify the amount of muscle activity the EMG integrals were computed from the EMG onset (t_0_) to the maximum peak. The integrated values were then normalized to the absolute maximum of the integrals across all series for each subject and for each muscle.

### Statistics

Repeated measures ANOVA was performed for each outcome variable considering the three groups Older (O), Younger adults (Y) and Skaters (S) as between factor. Pairwise comparisons with Bonferroni corrections were used to explore significant effects. A significance level α = 0.05 was used in all tests. Since the assumption of normal distribution could not be fulfilled for the drawing test, a Kruskal-Wallis non-parametric test for independent samples was performed to highlight any differences between groups.

## Results

### Analysis of the control conditions:

The individual postural stability was first analyzed in the control conditions. A repeated measures ANOVA considering the signals recorded from both force plates and the no-sound and brown noise as a within-subject factors, meanwhile the three population groups (O, Y and S) as between-subject factors did not give significance of the results. This analysis showed that no participants presented significantly different postural stability in presence of silence or uninformative noise, irrespectively of their age and motor skill level.

### Analysis of the normal force during the jump

**Figure 3:**
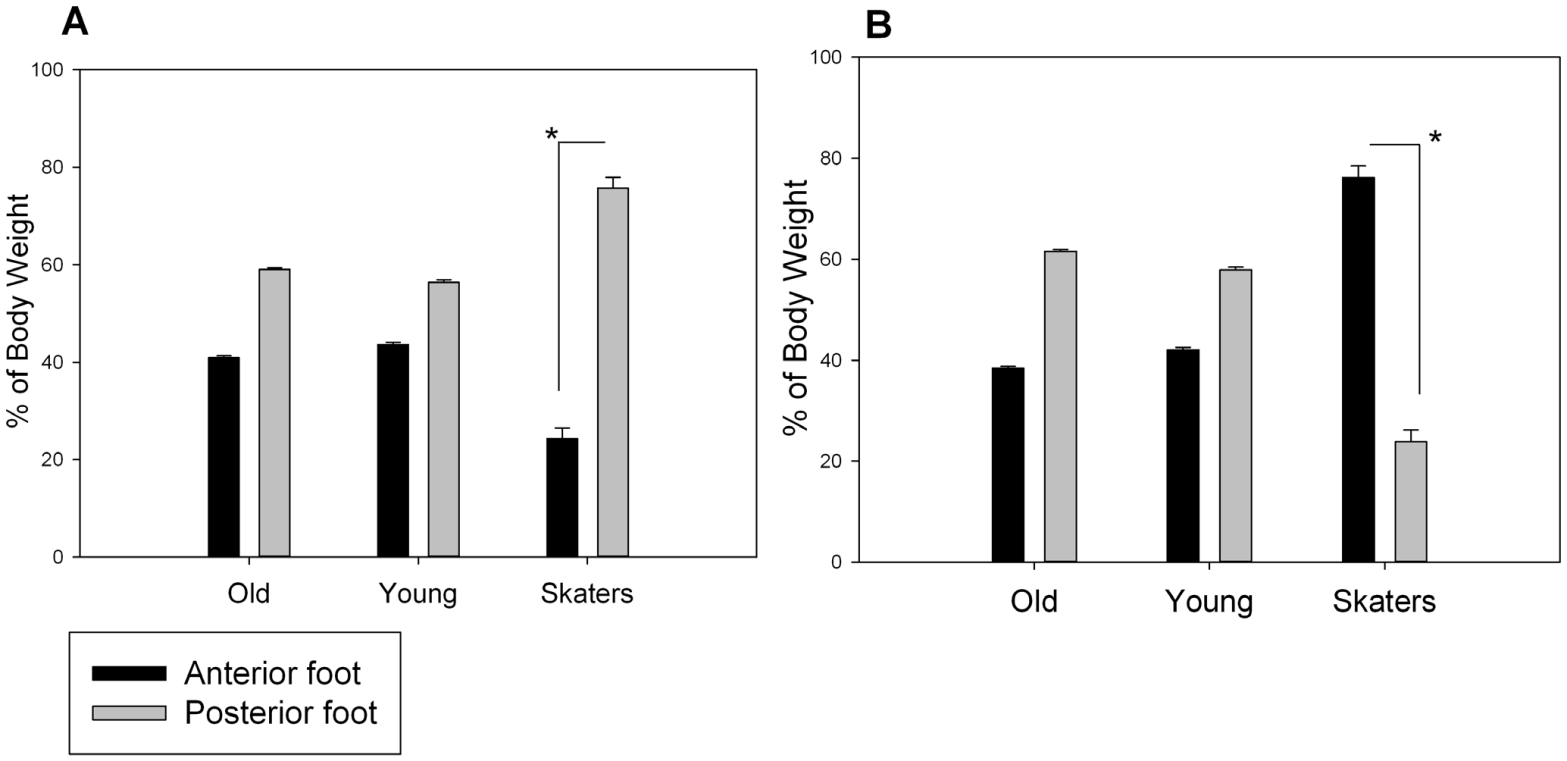
Means and standard deviations of the percentage of the total body weight produced during the jump by the three groups in the two force platforms (black bars represent the anterior and gray bars the posterior platform). Left graph shows the time to peak detected during the takeoff while right graph the time to peak detected during the landing phase. The * symbol indicates the level of significance <0.05.

For defining the dynamical postural strategy adopted during the jump simulation a 3×2×2 ANOVA with repeated measurements was performed for the normal force Fz in the two platforms considering Group O, Y and S as between subjects, and the two instants and the peak force Amplitude in the front and rear platform, respectively as within factor. The results showed a significant main effect of the Amplitude (F(1,17) = 22.126, p<0.0001, η^2^ 0.533). In general, participants applied more force on the rear platform. Significant interactions were found as well: Group×Amplitude (F(2,17) = 7.091, p = 0.006, η^2^ = 0.455), Peak-Force-Instant×Amplitude (F(1,17) = 29.781, p<0.0001, η^2^ = 0.544), Amplitude×Peak-Force-Instant×Group (F(2,17) = 26.027, p<0.0001, 26.027, η^2^ = 0.77). A post hoc for the first interaction, i.e. Group×Amplitude, considered the amplitudes of the peak forces of each platform together, and showed that while older and young adults presented an unbalanced distribution of the total force between platforms, conversely skaters equally distributed the forces between the two platforms. For the second interaction, i.e. Peak-Force-Instant×Amplitude, the post hoc showed that while in the first peak the front platform was loaded with less force than the rear one, in the second peak the opposite situation occurred. The triple interaction was separating the percentage of force produced by the peak for each force platform, and across groups. The post hoc revealed that, while for older and younger adults the force distribution in the two instants was almost equal across the two platforms, conversely a clear difference was present for skaters. In fact, skaters loaded the front and rear platform respectively with 24.27% and 75.72% of the total force during the first peak simulating the take off (Figure 3A), while exerting the opposite load during the second peak simulating landing (76.18% front and 23.81% rear) (Figure 3B).

### Analysis of EMG signals during the jump

**Figure 4:**
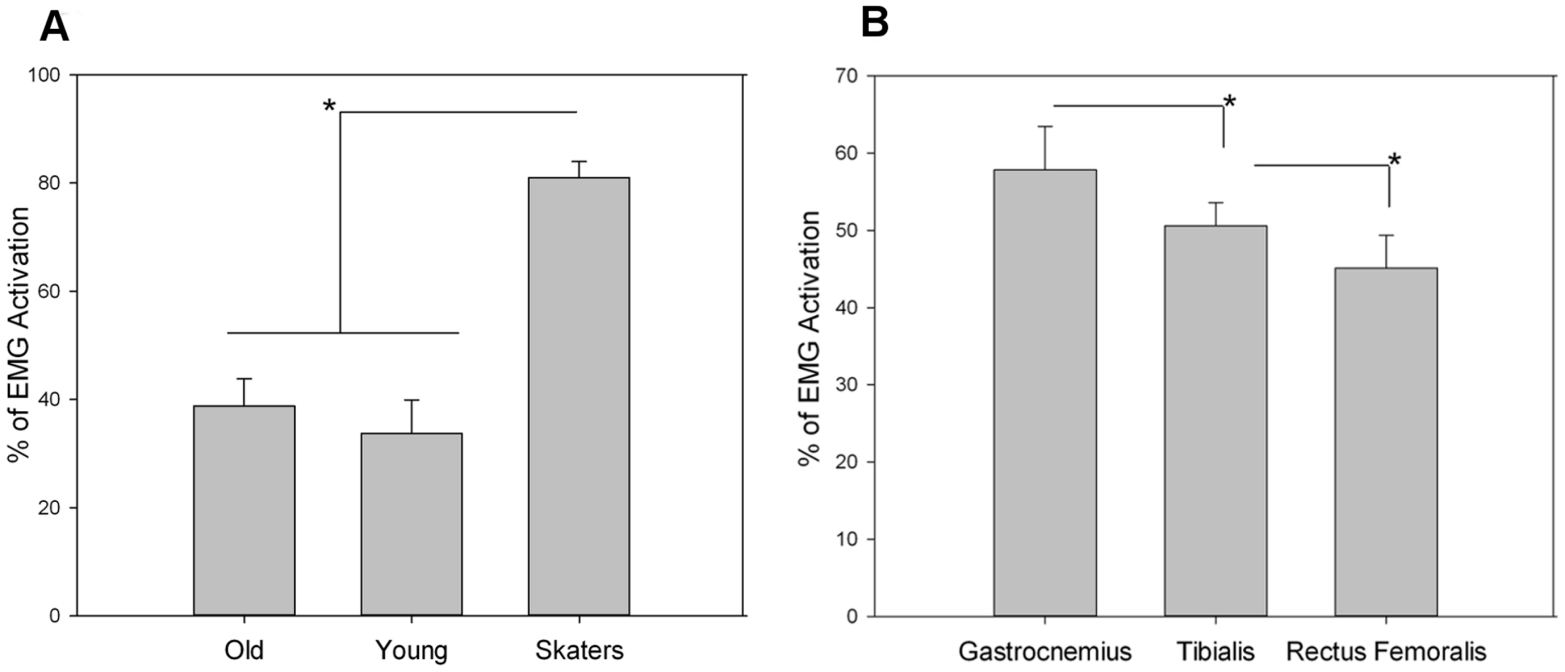
Means and standard deviations of the EMG magnitude expressed in percentage over the baseline: A) across groups and B) across the three lower legs muscles. The * symbol indicates the level of significance <0.05.

We performed an ANOVA (3×3×2) with repeated measures between the three groups, by considering the EMG data respectively from the G, TA, and RF muscle and the right and left part of the body. The between-subject factor was found to be significant: F (1,17) = 74.43, p<0.001, η^2^ = 0.864. The post hoc revealed that while older and younger adults presented similar muscle contraction above the baseline, skaters presented significantly higher values for all muscles activity (mean for O equal to 33.698, for Y equal to 38.803 and for S equal to 81.019) (Figure 4A). Moreover, there was a significant main effect for muscles (F (2,16) = 7.988, p = 0.0001, η^2^ = 0.285), showing the Gastrocnemius (G) to be the most active muscle, followed by Tibialis (TA) and finally the Rectus Femoralis (RF) (Figure 4B). We also found significance of the triple interaction Muscle×Body part×Group (F (4,34) = 2.838, p<0.05, η^2^ = 0.178). This interaction showed that O and Y presented unbalanced muscle contraction for the left part of the body: in particular, O presented higher contraction for G compared to TA (p = 0.007) and to RF (p<0.001); Y subjects with the same trend presented significant differences between G and RF (p = 0.002). S subjects instead presented perfect balance among muscles for this part of the body. For the right part of the body all the participants presented a balanced amount of contraction among muscles.

### Analysis for action preparation (APAs)

**Figure 5:**
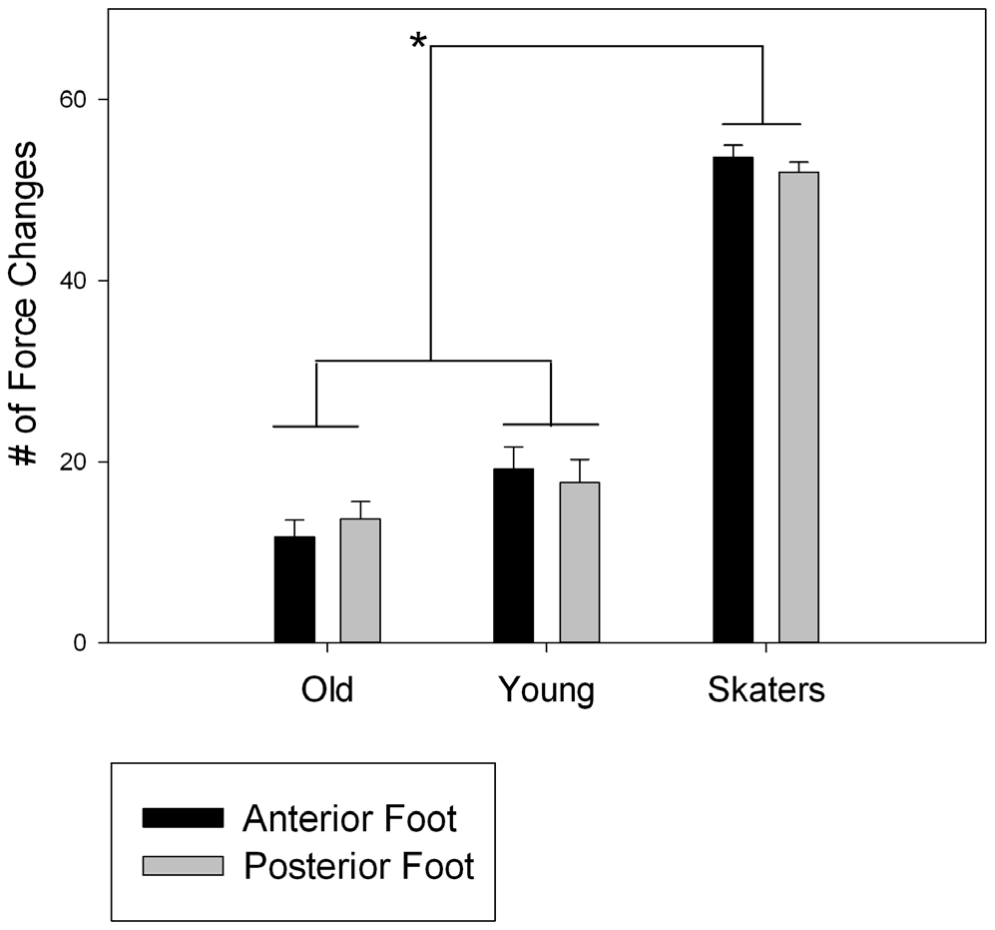
Number of force changes detected immediately before and after the initiation of the sound of the jump for the three groups and in each force platform. The * symbol indicates the level of significance <0.05.

We counted the number of frontal-rear and lateral-lateral instants of change of forces within the time window [t_0_−300, t_0_+300] ms and transformed in percentage of the total number of trials. Since a first analysis showed no differences in the force modulations in the x and y directions, we considered their mean, and considered, for each subject and platform, how many trials presented a force change. An ANOVA (3×2) with repeated measures was performed between the three groups, considering the percentage of force changes present in the frontal and rear platform as within factor. The results did not show a significant effect for the platform (F (1,17) = 0.375, p = 0.754, η^2^ = 0.005), suggesting that no significant differences existed between the frontal and rear foot. Conversely, a significant difference was found between subjects (F (1,17) = 53.806, p<0.0001, η^2^ = 0.863). A post hoc test revealed that differences exist between S and O (p<0.0001), and between S and Y (p<0.0001), whereas no differences were found between O and Y (p = 0.678) (Figure 5). On average, skaters showed APAs 88.02% of the time, while older and young adults showed APAs respectively 21.11% and 30.69% of the time. Since only the skaters exhibited a significant number of force APAs immediately before as well as after the jump event, in this case we computed the time when such changes appeared on both force platforms. No significant difference was found between the front and rear platform (p = 0.589), showing that skaters moved their feet simultaneously. On average, APAs appeared at t_0_+220 ms and at t_0_+200 ms respectively in the anterior and in the posterior foot.

### Drawing test

**Figure 6:**
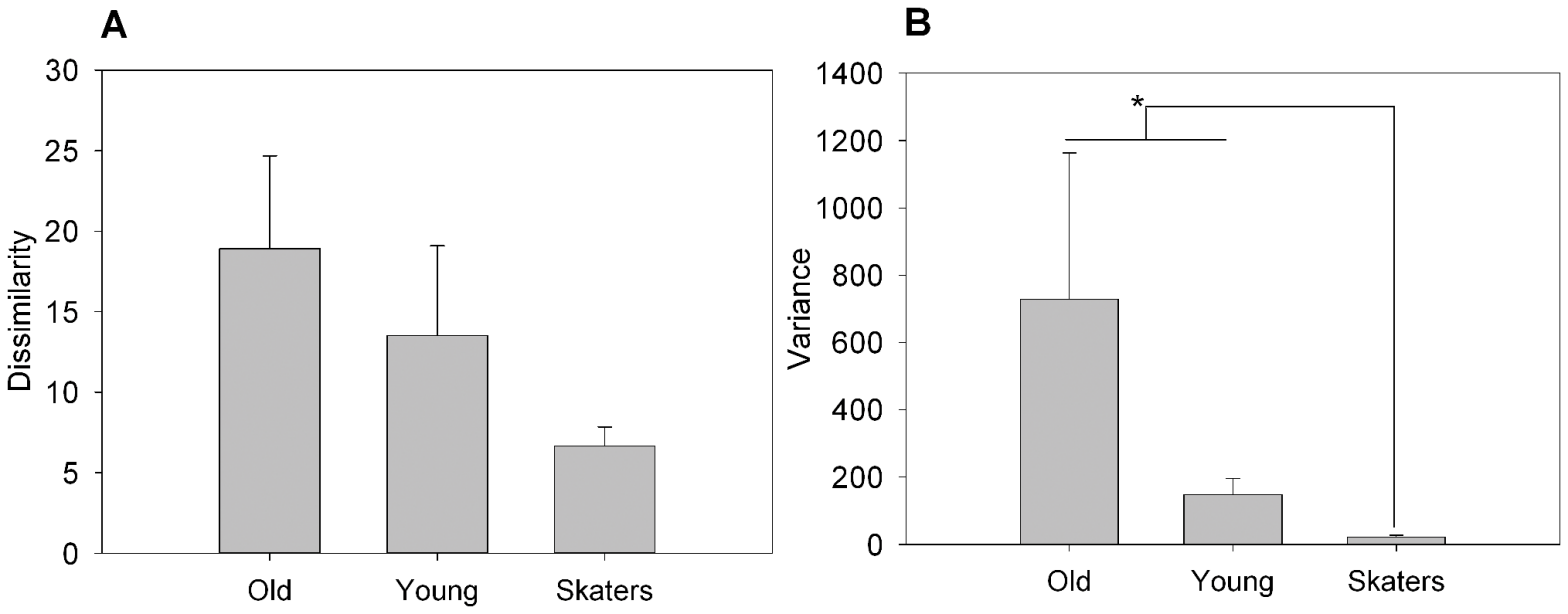
A) Mean and standard deviation for the drawings dissimilarity among groups. B) Variance and standard deviation of the variance for the drawings dissimilarity among groups. The values are defined by the Dynamic Time Warping (DTW) procedure, divided by 1000. Higher scores represent lower draw precision and the * symbol indicates the level of significance <0.05.

**Figure 7:**
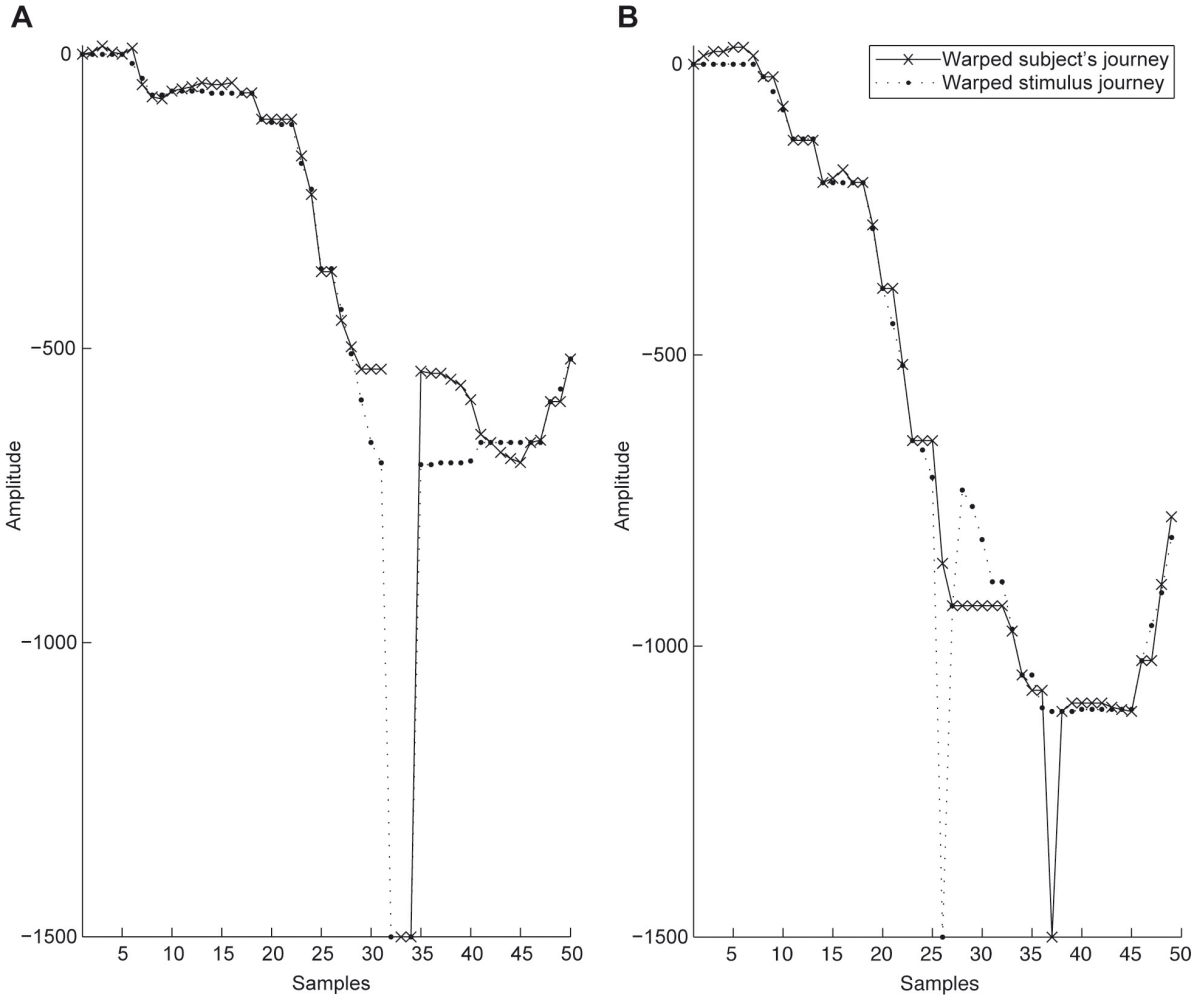
Good alignment A) and poor alignment B) between the prescribed (dot symbols) and the drawn (cross symbols) traces, after Dynamic Time Warping (DTW).

In order to test the ability to correctly recall the experienced sound (Sound S1 and Sound S2), we asked participants to draw the altimetry profiles of the travel on a paper sheet as explained in more details in the Appendix S1. We performed a Kruskal-Wallis non-parametric test for independent samples. Results showed no difference between S and O, between S and Y, and between Y and O (p>0.05, Cohen's d = 4.92) indicating that all subjects were able to represent the experience with comparable precision (Figure 6A). The same non-parametric test was then performed but this time considering the variance of the drawn altimetry values across trials. The results showed a difference between S and O (p<0.05, Cohen's d = 4.92) and between S and Y (p<0.05, Cohen's d = 1.53), while Y and O showed no significant difference (Figure 6B and Figure 7 for exemplar data).

## Discussion

In this study we were interested to unravel whether humans are prompt to anticipate and to reproduce a skateboarding jump just by listening to the sound it produces, and to explore whether the level of familiarity and motor skills that listeners have in sport in general and in skateboarding in particular would affect this ability. We showed that only skaters modulated the forces underfoot, and enacted muscle synergies that closely resembled the ones that a skater would apply if actually jumping on a skateboard. On the contrary, old and young adults presented forces and muscle activations primarily aimed at maintaining balance stability. More importantly we showed that only skaters act an online control of the movement by initiating the jump simulation early on at around 200 ms after the start of the jump event (Figure 2). All individuals, independently from their age and expertise, recalled with similar precision the paths experienced through the sound, but only skaters were highly reproducible in their recollection.

It is important to remind that the task for each individual was to simulate the run on a skateboard, by listening to a sonic sequence of events that evoked different moments of the action: acceleration, deceleration, constant velocity and jump. We focused on the jump event to investigate on the individual ability to anticipate and simulate a complex and tight perception-action process. Jump event was 1500 ms long: across this time, individuals should take into account both take off and landing to reproduce a coherent action. We found that skaters were able to simulate a pattern of action that amazingly resembled the one that they would apply if actually jumping. In fact, skaters follow the biomechanics of a real jump by displacing their body weight (BW) backward during take off, and then shifting the BW forward across their flight to prepare for landing and touching the ground first with the forward foot [45]. By listening to the sound, skaters simulated a remarkably comparable pattern of motion as it is performed during an actual skateboard jump by shifting their body weight from backward to forward (see Figure 3). Furthermore, it is striking that such dynamic changes in BW distribution were simulated in such a limited time. On the contrary, the other groups were presenting a completely different strategy as they kept most of their BW firmly on the rear foot across the whole jump event. As a general rule, while skaters were proactive in following the sound, young and older adults appeared to be more conservative in their motor strategy as if the sound was perceived as a source of posture destabilization.

It is worth noticing that on one side young non-skaters, although being highly active in several sports, were not able to act as skaters did, while on the other side young and older non-skaters did not differ in their ability to simulate the skateboarding action. Our results showed that, in spite of the fact that all subjects were able to recognize the sound and the represented action phases, different motor strategies were found when comparing the skaters with the two non-skaters groups. Conversely no differences were found between young and older non-skater adults, showing that action recognition and action simulation are motor skills and age independent. Interestingly, differences in action performance appeared in spite of the fact that all three groups showed similar precision in drawing the paths. This suggests that the auditory recognition of an action does not strictly depend on the level of motor ability possessed by the listener [46], [47]. In the visual system, two cortical pathways have been already identified which integrate their respective function: the former is slower but long-lasting, and located in the ventral system so as to facilitate object recognition and identification; the latter, located in the dorsal side, using instantaneous visual information for a fast and continuous control of the actions [48]. Like in the visual domain, distinct neural pathways might be necessary: one for sound identification, and one for dynamic action control based on auditory perception [49]–[51]. Here, we suggest the presence of distinct neural pathways: one for sound recognition, and one for continuously controlling the action based on auditory recognition.

The presence of an internal action simulation was also supported by the muscular activations measured during the task. Again, following the biomechanics of the jump, during take off and landing skaters apply an amount of force that is usually 2–3 times the individual BW [45]. Indeed, in our experiment skaters applied a larger amount of muscle contraction compared to the other two groups.

### Conclusions

Skaters presented consistent Anticipatory Postural Adjustments (APAs) immediately after (on average 200 ms) the beginning of the auditory event, indicating their ability to use the sound information to deal with the jump and plan the action. On the contrary, for young and older non-skaters APAs were present discontinuously. While the presence of APAs between 50 to 300 ms after a (usually visual) ‘go’ signal has been shown in many occasions [35], [52], [53], here for the first time we show that APAs can be triggered by the sound produced by an action [24]–[26] and that their modulation can be refined through sport practice.

## Supporting Information

Appendix S1 **Dynamic time warping (DTW - dtw routine) used to compute the distance (or dissimilarity) between each trace.**
(DOCX)

Sound S1 **Trace of Sound Stimuli.**
(WAV)

Sound S2 **Trace of Sound Stimuli.**
(WAV)
