# Correction: Might as Well Jump: Sound Affects Muscle Activation in Skateboarding

**DOI:** 10.1371/journal.pone.0100976

**Published:** 2014-06-16

**Authors:** The PLOS ONE Staff

There is an error in Reference 19. The correct reference is:

Turchet, L., Serafin, S., & Cesari, P. (2013). Walking pace affected by interactive sounds simulating stepping on different terrains. ACM Transactions on Applied Perception (TAP), 10(4), 23:1-23:14
