# Supplementary material for: Might as Well Jump: Sound Affects Muscle Activation in Skateboarding
Source: PLoS One. 2014 Mar 11;9(3):e90156. doi: 10.1371/journal.pone.0090156 (PMC3949725; doi:10.1371/journal.pone.0090156)
Supplement: Appendix S1 — Dynamic time warping (DTW - dtw routine) used to compute the distance (or dissimilarity) between each trace. (DOCX) [file pone.0090156.s001.docx]

**S1**

Each subject provided four drawings, labeled by probe number n = {1, 2}, and trip number v = {1, 2}. Drawing was done on paper having a square grid with 5 − mm cells printed on it (see Figure 1B). Pages were scanned and acquired as digital images.

These images were passed to a Matlab routine for manual sampling through the ginput function (see Figure 1B). The jump was manually identified by a double click on each of its boundary points. The results were sequences of points (here called traces), each with its x and y coordinates, where double points indicate the jump. Traces were normalized to have the leftmost point with y coordinate at 0, and to have the jump represented by a rectangular dip whose bottom is at y = −1500.

Dynamic time warping (DTW - dtw routine) was used to compute the distance (or dissimilarity) between each trace and the corresponding nominal trip, similarly represented as a sequence of points. DTW has been widely used for the temporal alignment of signals, especially in speech processing. It has also been used for matching spatial shapes, as in the case of signature verification . For the purpose of this analysis, it makes sense to further normalize the vertical extension of traces before applying DTW. In particular, we linearly scaled the vertical coordinate in such a way that each trace, starting from y = 0, has a minimum that is the same as that of the corresponding nominal trip trace. This scaling is done without considering the jump, which keeps being represented by points at y=−1500. DTW finds an optimal match between the drawn and prescribed traces by allowing for local stretching and compression along the x-axis. Therefore, the exact x coordinates of each point in the trace become irrelevant for DTW, as the processed trace is just an ordered sequence of y coordinates. In most of the cases, for drawn traces that resemble the stimulus traces, the jump gets aligned between the drawn and stimulus traces (see Figure 7A). In Figure 7 panel B there is a case of matching where the drawn jump is not aligned with the stimulus jump. The dissimilarity measures returned by DTW were used.
